# Visual working memory guides attention rhythmically in humans

**DOI:** 10.7554/eLife.108017

**Published:** 2026-07-08

**Authors:** Jiachen Lu, Yaochun Cai, Xilin Zhang

**Affiliations:** 1 Department of Psychology and Research Center of Adolescent Psychology and Behavior, School of Education, Guangzhou University, Guangzhou, China Guangzhou China; 2 Key Laboratory of Brain, Cognition and Education Sciences, Ministry of Education, South China Normal University Guangzhou China; 3 School of Psychology, South China Normal University Guangzhou China; 4 Center for Studies of Psychological Application, Guangdong Key Laboratory of Mental Health and Cognitive Science, South China Normal University Guangzhou China; 5 Philosophy and Social Science Laboratory of Reading and Development in Children and Adolescents, Ministry of Education, South China Normal University Guangzhou China; Washington University in St. Louis United States; Peking University China

**Keywords:** visual working memory, visual attention, Rhythmic-Item-Template, theta oscillation, alpha oscillation, Human

## Abstract

How does internal representation held in visual working memory (VWM), known as the attentional template, guide attention in humans? A longstanding debate concerns whether only one (Single-Item-Template theory) or multiple (Multiple-Item-Template theory) items serve as attentional templates simultaneously. Here, we propose a Rhythmic-Item-Template hypothesis, successfully reconciling these seemingly contradictory theories. Using the classical VWM-guided attention task with human participants, we found that two VWM items alternately dominate behavioral guidance in theta-rhythmic (4–8 Hz), with anti-correlated activation states in time, and more importantly, this rhythmic oscillation was not driven by the retro-cue processing. Neural recordings revealed that occipital alpha oscillation (8–14 Hz) governed item-specific prioritization, and its amplitude closely tracked subjects’ behavioral guidance, while frontal theta-oscillations phase-led and coupled with occipital alpha oscillations during the item transition. Our Rhythmic-Item-Template results not only resolve previous Single-Item-Template versus Multiple-Item-Template debate but also advance our understanding of how distributed brain rhythms coordinate flexible resource allocation in multi-item memory systems.

## Introduction

The capacity of visual working memory (VWM) to guide attention through multiple internal templates remains contested (Frătescu et al., 2019). While VWM can store several items (Constantinidis and Klingberg, 2016; Cowan, 2001; Oberauer et al., 2016), behavioral studies conflict: some suggest only one item dominantly guides attention at any moment (Büsel et al., 2019; Olivers et al., 2011), whereas others report parallel guidance by multiple templates (Kerzel and Witzel, 2019; Kristjánsson and Kristjánsson, 2018). Hollingworth and Beck, 2016, revealed that distractors matching either of two target colors elicited equivalent attentional capture, supporting dual templates. However, attenuated capture magnitude compared to single-item conditions exposed a critical paradox: if multiple templates coexist, why does their behavioral efficacy diminish? Three hypotheses emerge: (1) transient dominance of a single item suppresses others, (2) independent but weakened template influences, or (3) rhythmic alternation between items via theta band oscillations (4–8 Hz). Critically, the third hypothesis posits that limited attentional resources are dynamically allocated through cyclical prioritization rather than static competition—a mechanism aligning with the oscillatory nature of neural processes (Fiebelkorn et al., 2018; VanRullen, 2016).

This oscillatory framework is rooted in attention’s discrete temporal dynamics. When monitoring two locations, human attention rhythmically samples each location at 4–10 Hz, with asynchronous peaks between sites—a ‘sequential attentional spotlight’ (Landau and Fries, 2012). Neural recordings demonstrate that alpha band suppression (8–14 Hz), reflecting attentional engagement, alternates between objects every ~200 ms (theta rhythm; Jia et al., 2017). These findings imply a conserved theta-rhythmic mechanism for resolving representational competition, whether for external stimuli or internal VWM representations. Recent studies extend this to VWM: multi-item retention exhibits 6–10 Hz oscillatory patterns (Chota et al., 2022; Peters et al., 2021; Pomper and Ansorge, 2021), yet debates persist about whether these rhythms reflect true prioritization dynamics or spatiotemporal confounds (Huang et al., 2018; Liu et al., 2019).

Moreover, previous evidence for rhythmic memory processing primarily stems from behavioral measures, lacking direct neural evidence of stimulus-specific modulation. While alpha oscillations (8–14 Hz) in visual cortex track VWM item prioritization (de Vries et al., 2017; Pavlov and Kotchoubey, 2022; Wolff et al., 2017), they likely enhance signal-to-noise ratios through selective neural recruitment rather than directly governing task goals (Benedek et al., 2014; Klimesch, 2012; Peylo et al., 2021). Conversely, frontal theta oscillations (4–8 Hz) coordinate goal-directed behaviors and task switching (Helfrich et al., 2017; Josselyn and Tonegawa, 2020), exerting top-down control over sensory regions via phase synchronization (Berger et al., 2019; van Driel et al., 2017). Critically, fronto-posterior network coupling mediates control shifts in VWM: increased frontal theta power predicts contralateral occipital alpha suppression during priority transitions (Audrain et al., 2020; de Vries et al., 2020; Vries et al., 2018). This supports a mechanistic hypothesis: frontal theta oscillations rhythmically drive spatially distributed alpha oscillations through cross-frequency coupling, dynamically activating multiple VWM items in theta-rhythmic cycles.

To test this theoretical proposition, we designed and conducted three experiments using a memory-search paradigm that effectively rules out strategic control (Soto et al., 2007). Experiment 1, with dense temporal sampling, revealed 7 Hz anti-phasic oscillations in attentional capture between two VWM items. Experiment 2, by implementing individualized temporal analysis to eliminate retrospective cueing artifacts, replicated the 7 Hz behavioral rhythm. To further explore the neural mechanisms and comprehensively validate our theoretical hypothesis, we carried out Experiment 3, in which electroencephalography (EEG) was employed to record and analyze brain electrical activity with high temporal resolution. EEG results demonstrated that activation in the occipital alpha band was closely associated with behavioral performance and exhibited significant phase coupling with prefrontal theta oscillations. Additionally, during the memory maintenance phase, the coupling strength of alpha-theta between the bilateral visual cortex and the prefrontal cortex alternated in a theta-rhythmic pattern in leading. These results collectively establish frontally driven theta-alpha coupling as a mechanism for rhythmically allocating attentional access to multiple VWM items, reconciling the divergence between single- and dual-template theories through a dynamic, oscillation-based model.

## Results

### Experiment 1: Theta-rhythmic oscillation of VWM

**Figure 1.**
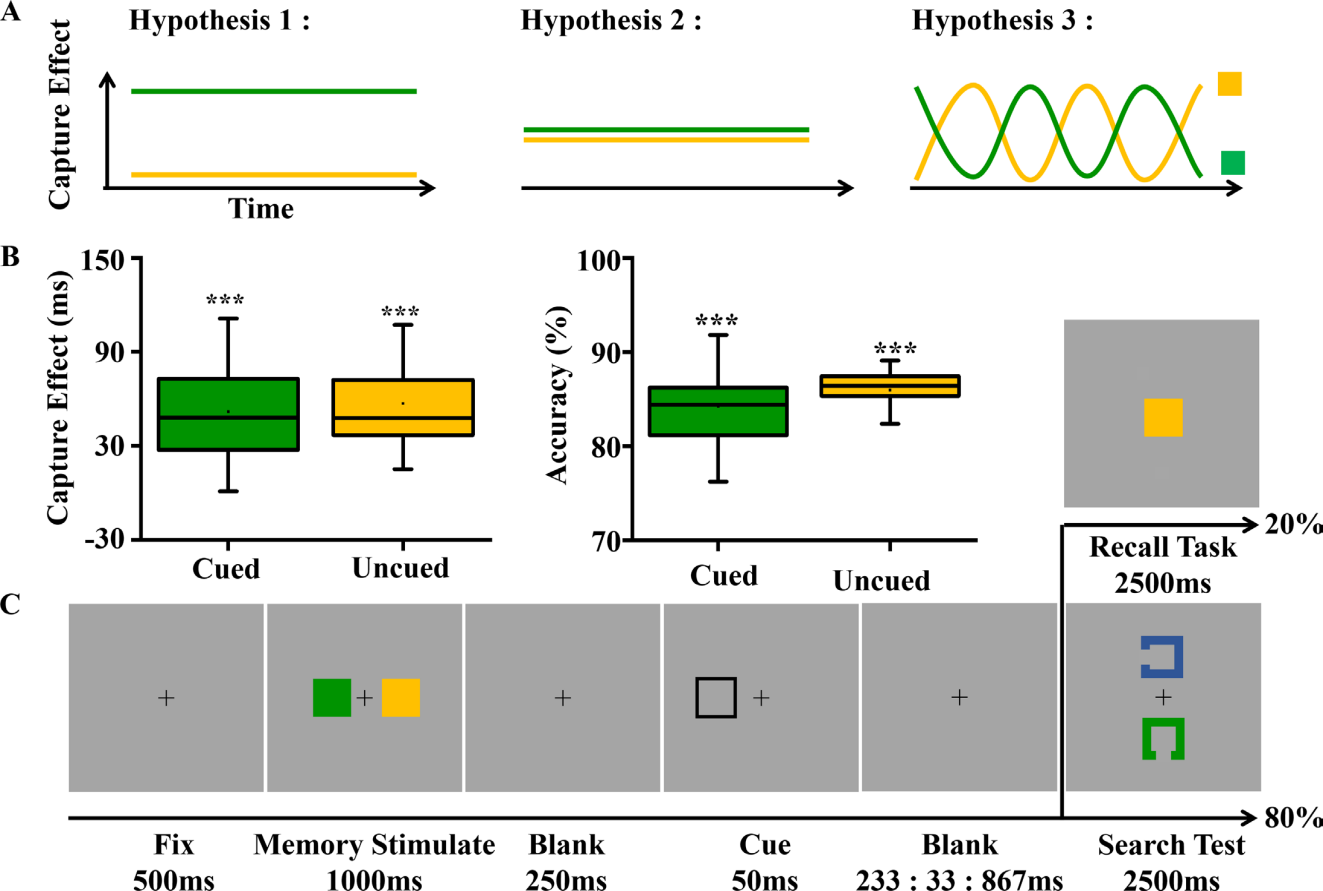
Hypothetical models, experimental paradigm, and behavioral results. (**A**) Hypothetical models illustrating the activation patterns of two memory items during the memory retention phase. Hypothesis 1 posits a single template, Hypothesis 2 suggests multiple templates, and Hypothesis 3 proposes dynamic templates. (**B**) Behavioral results, showing the attentional capture effect size (calculated as the difference in response time between the distractor matching the memory items and the target matching the memory items) and memory accuracy for the two memory items. n = 25. (**C**) Experimental procedure. A cue stimulus randomly indicated one of the two memory items. In 80% of the trials, participants performed a search task to identify the item with a gap facing upward or downward. In 20% of the trials, participants performed a recall task to determine whether the probed item matched one of the two memory colors.

25 participants engaged in Experiment 1. As shown in Figure 1C, subjects were asked to remember two memory items that appeared simultaneously. During the memory retention interval, a cue randomly instructed to one of the items, directing internal attention toward the cued stimulus. To examine the time course of VWM, the response interface appeared at various time intervals following the cue, with a high temporal resolution (stimulus onset asynchrony [SOA]: starting at 233 ms and increasing in increments of 33 ms up to 867 ms). The response interface required participants to complete either a search task (80% of trials) or a recall task (20% of trials). In the search task, participants were asked to search a target square (with a gap at the top/bottom) while disregarding a distractor square (with a gap on the left/right). To ensure the search target and the memory item did not share the same spatial location, the target and distractor were positioned vertically relative to the participants’ point of gaze. Only one of the target and distractor matched the color of memory items, or neither did. The attentional capture effect was measured as the difference in response time between the distractor matching the memory items and the target matching the memory items. In the recall task, participants were asked to determine whether a presented color corresponded to one of the memory items, thereby assessing the accuracy of item retention.

Initially, we calculated the recall accuracy for both memory items, as depicted in Figure 1B. The recall accuracy for cued items (t(24) = 91.60; p<0.001, Cohen’s d=37.39) and uncued items (t(24) = 70.74; p<0.001, Cohen’s d=28.88) significantly exceeded the chance level (0.5), demonstrating effective memory retention for both item types. Subsequently, we examined the attentional capture effects associated with these memory items. The analysis revealed that the magnitude of the attentional capture was significantly above zero for both cued items (t(24) = 6.16; p<0.001, Cohen’s d=2.51) and uncued items (t(24) = 8.02; p<0.001, Cohen’s d=3.27), indicating that both items effectively captured attention. These findings align with previous studies (Hollingworth and Beck, 2016), confirming that the human brain is capable of retaining multiple memory items simultaneously, with each item significantly influencing attentional processes.

**Figure 2.**
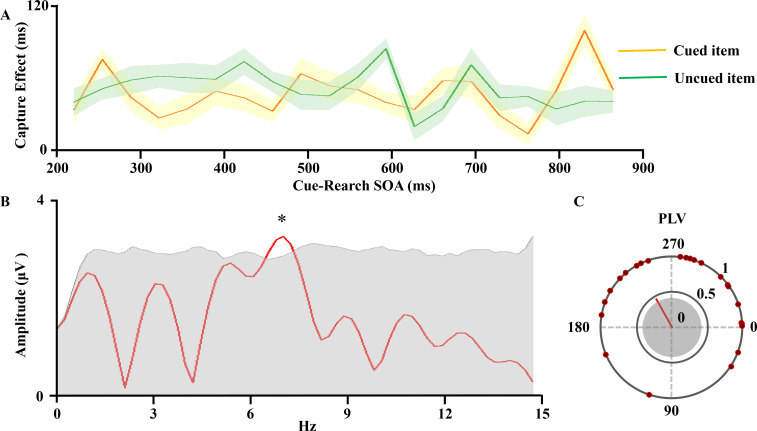
Rhythmic alternation at 7 Hz and anti‑phasic relationship of attentional capture effects. (**A**) Line graph. The attentional capture effect size for the cued and uncued items across different time intervals (stimulus onset asynchrony [SOA]), calculated as the difference in response time between the invalid and valid conditions. (**B**) Spectrum plot. The red line represents the amplitude of the values from A at different frequencies; the gray line indicates the 95th percentile from the permutation test; *: p<0.05. (**C**) Phase-locking value (PLV). The red line shows the PLV at 7 Hz for the cued and uncued items, representing the average phase difference across all participants; gray circles indicate the 0–95 percentile range from the permutation test; blue hollow circles represent the phase differences for individual participants between the two items.

With respect to the central question of our study, we found clear evidence for memory item sampling as a preferred template. As shown in Figure 2A, we examined the attentional capture effects of cued and uncued items separately for different temporal SOA conditions. It was found that the capture effects of these two items alternated in dominance rather than remaining stable (F(1, 19)=4.67, p<0.001, η2=0.16). Specifically, the capture effect of cued items was significantly greater than that of uncued items at SOAs of 267 ms (t(24) = 2.72, p=0.03, Cohen’s d=1.11), 667 ms (t(24) = 2.37, p=0.03, Cohen’s d=0.97), and 833 ms (t(24) = 3.53, p=0.002, Cohen’s d=1.44), while the capture effect of uncued items was significantly greater than that of cued items at SOAs of 333 ms (t(24) = 2.97, p=0.007, Cohen’s d=1.21), 367 ms (t(24) = 2.14, p=0.04, Cohen’s d=0.87), 433 ms (t(24)=2.49, p=0.02, Cohen’s d=1.02), 467 ms (t(24)=2.37, p=0.03, Cohen’s d=0.97), and 567 ms (t(24)=2.72, p=0.02, Cohen’s d=1.11).

In addition, as shown in Figure 2B, the Fourier transformation was applied to the time course of the item-based benefit to determine the temporal frequency of these fluctuations. The item-based benefit was calculated as the difference in capture effects between the cued and uncued items for equidistant time intervals ranging from 200 ms to 833 ms. The amplitude spectrum analysis of frequencies ranging from 1 Hz to 15 Hz (Figure 2B) revealed a significant peak at 7 Hz (p<0.05, FDR corrected). This finding implies that the rhythmic fluctuations in attentional capture associated with VWM are likely driven by an oscillatory mechanism within the theta frequency range, supporting the proposed sampling frequency in VWM.

Consistent with the hypothesis that only a single memory item serves as a prioritized template at any given moment, the speed at which cued and uncued items are accessed should exhibit a negative correlation, as demonstrated in previous research on visuospatial and object-based attention (Landau and Fries, 2012; Fiebelkorn et al., 2013). To investigate the possibility that attention within VWM alternates between items, we analyzed the Fourier coefficients at 7 Hz corresponding to the attentional capture effects for both cued and uncued items and subsequently calculated their phase-locking values (PLV; Fiebelkorn et al., 2013). As shown in Figure 2C, the phase-locking between the 7 Hz rhythms of the two items was found to be significant (PLV = 0.402, p<0.05), displaying an average phase angle difference of 118° (95% CI from 48° to 188°). This anti-phase relationship between the oscillations supports the notion that internal attention alternately samples items in working memory.

### Experiment 2: Behavioral oscillation without cueing

**Figure 3.**
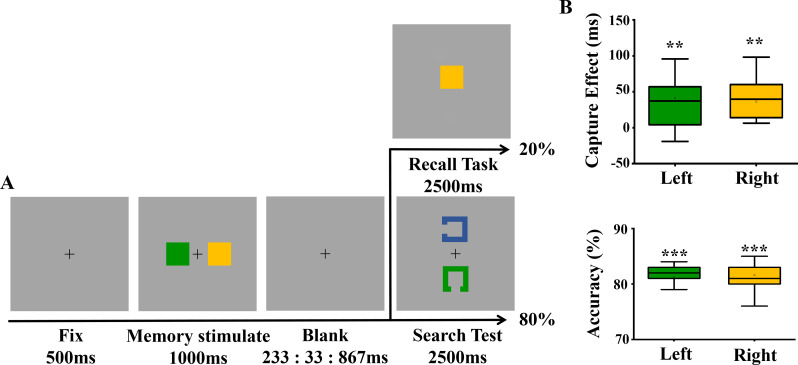
Experimental paradigm and behavioral results without retro‑cue. (**A**) Experimental procedure. Two memory items are presented simultaneously without any post-cue prompts. In 80% of the trials, participants performed a search task to identify the item with a gap facing upward or downward. In 20% of the trials, participants performed a recall task to determine whether the probed item matched one of the two memory colors. (**B**) Behavioral results, showing the attentional capture effect size and memory accuracy for the two memory items. n = 17.

17 participants took part in Experiment 2, which closely replicated the design of Experiment 1 with one critical modification: no retro-cue was presented after the two memory items. Instead, the probe appeared directly after one of three fixed SOAs (233:33:867 ms; see Figure 3A). This adjustment was implemented to rule out the possibility that working memory oscillations were driven by cue processing rather than intrinsic maintenance mechanisms.

As illustrated in Figure 3B, the recall accuracy for both the left memory item (t(16) = 53.40, p<0.001, Cohen’s d=13.35) and the right memory item (t(16) = 51.10, p<0.001, Cohen’s d=12.78) significantly exceeded the chance level (0.5), suggesting effective retention of the memory items by participants. Furthermore, our analysis of the attentional capture effect of both memory items revealed that the effect size was notably higher than zero for both the left item (t(16) = 3.24, p=0.005, Cohen’s d=0.81) and the right item (t(16) = 3.21, p=0.005, Cohen’s d=0.80), demonstrating that both memory items were effective in capturing attention.

**Figure 4.**
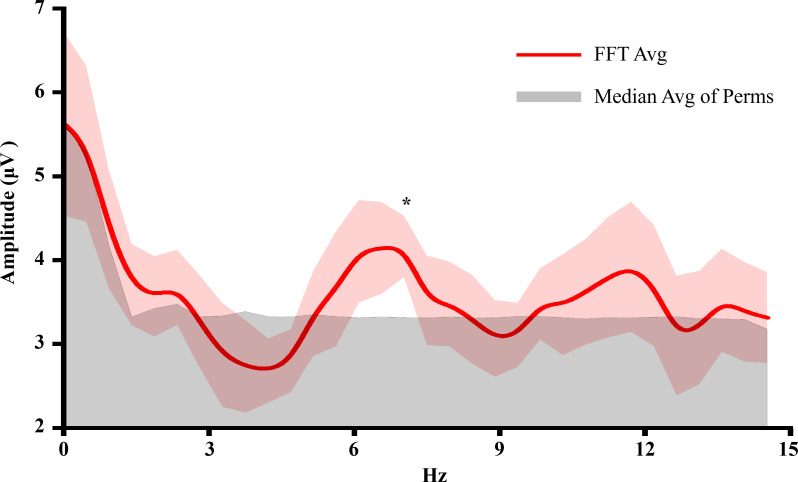
The red line represents the average across all participants of the Fourier transforms of the differences in capture effects between left and right memory items at the individual level. The gray area represents values below the group average of medians derived from 1000 permutations, with each permutation involving Fourier transforms for each participant. *: p<0.05.

Since no retro-cue was presented in Experiment 2, the data lacked a fixed temporal reference point across participants. Thus, unlike in Experiment 1 (where capture effects were group-averaged before Fourier analysis), we performed Fourier transforms individually for each participant before grand-averaging. Specifically, we computed the item-based benefit for each participant by subtracting the capture effect of the right memory item from that of the left item, evaluated at equidistant time intervals (233–867 ms). For each participant, we conducted amplitude spectrum analysis (1–15 Hz) on these time series. The resulting spectra were then grand-averaged across participants (red line, Figure 4). To assess whether spectral power exceeded chance levels, we generated a null distribution by (1) randomly shuffling each participant’s time series, (2) recomputing the Fourier transform, and (3) repeating this procedure 1000 times. The median amplitude of these surrogate data served as the chance baseline. A paired-samples t-test comparing the original amplitudes against this null distribution revealed significant oscillatory power at 7 Hz (p<0.05, FDR corrected).

### Experiment 3: Neural mechanisms of VWM oscillations

**Figure 5.**
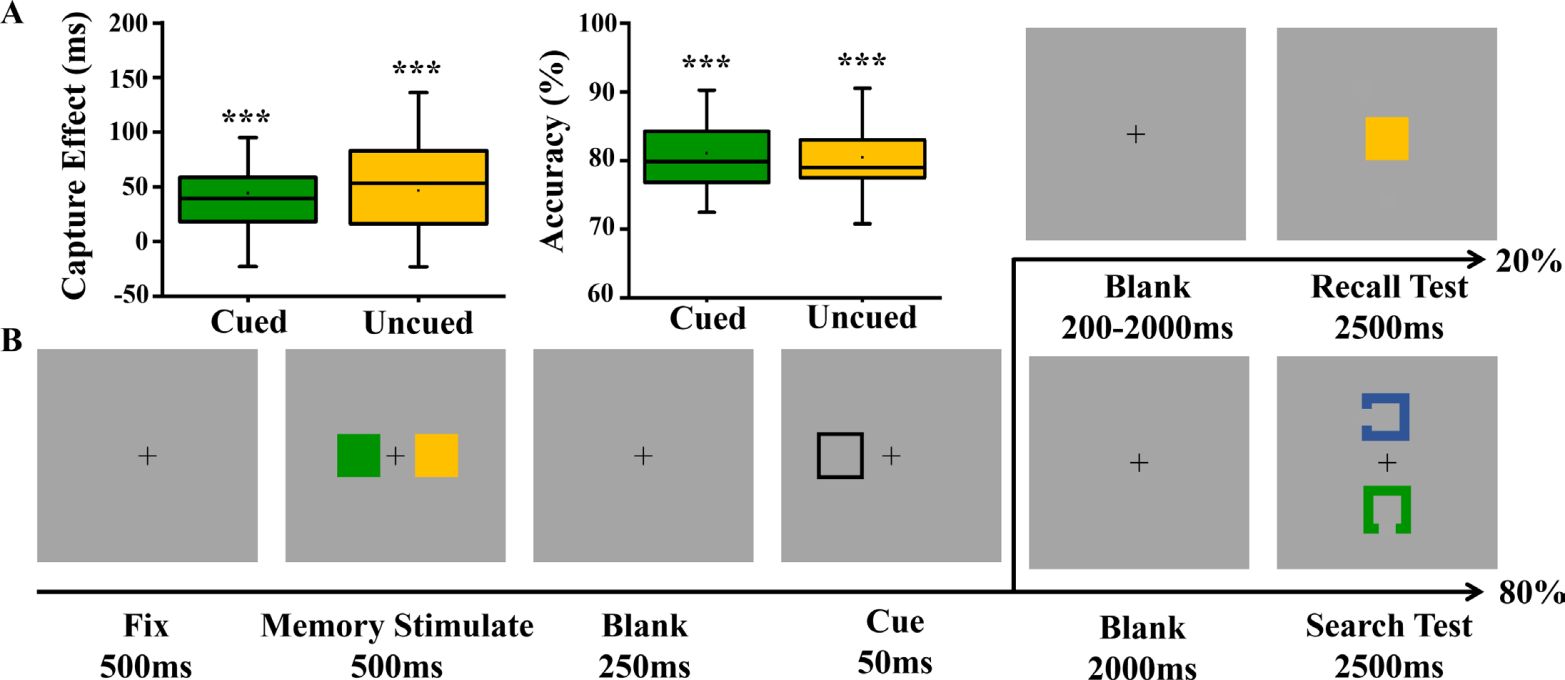
Behavioral results of Experiment 3. (**A**) Behavioral results, showing the attentional capture effect size and memory accuracy for the two memory items. n = 24.(**B**) Experimental procedure. A cue stimulus randomly indicated one of the two memory items. In 80% of the trials, participants performed a search task to identify the item with a gap facing upward or downward. In 20% of the trials, participants performed a recall task to determine whether the probed item matched one of the two memory colors.

In Experiment 3, 27 participants were recruited, with 3 participants excluded due to excessive artifacts in EEG data. This experiment differed from Experiment 1 in that the search task and the cue were presented at a fixed interval of 1500 ms, while the interval between the memory task and the probe varied randomly between 200 ms and 2000 ms (see Figure 5B). All other aspects of the experiment remained consistent with those of Experiment 1. Additionally, EEG data were recorded simultaneously from participants.

First, we calculated the recall accuracy for both memory items. As shown in Figure 5A, cued (t(23) = 45.70, p<0.001, Cohen’s d=19.06) and uncued items (t(23) = 23.56, p<0.001, Cohen’s d=9.82) were both recalled correctly at a significantly greater level than the chance level (0.5), indicating successful retention of both types of items. Additionally, we analyzed the attentional capture effects of the two memory items. The findings revealed that the attentional capture effects for cued (t(23) = 6.45, p<0.001, Cohen’s d=2.69) and uncued items (t(23) = 5.61, p<0.001, Cohen’s d=2.34) were significantly greater than zero, indicating that both types of memory items effectively captured attention.

**Figure 6.**
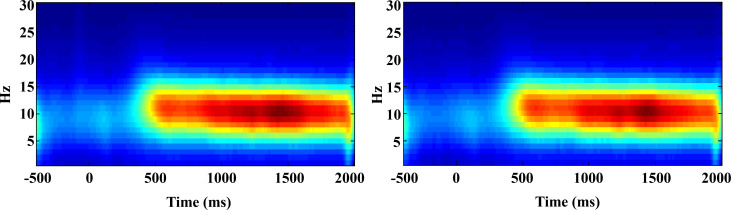
Time-frequency activation of contralateral occipital alpha-band (8-14 Hz) during memory retention. (Left) Time-frequency results of the contralateral occipital lobe (PO7/8) for cued items, showing significant alpha band activation (8–14 Hz) during the memory retention phase. (Right) Time-frequency results of the contralateral occipital electrodes (PO7/8) for uncued items, with a similar activation pattern to that observed for cued items.

Subsequently, an individual time-frequency analysis was conducted on the EEG data during the memory retention phase before the search task. This analysis delved into the temporal and spectral dynamics across frequencies from 1 Hz to 30 Hz and time intervals from –500 ms to 2000 ms for each participant. Consistent with prior studies (VanRullen and Macdonald, 2012; de Vries et al., 2017), pronounced alpha band activity (8–14 Hz) was observed in regions contralateral (PO7/8) to the memorized items (see Figure 6).

To evaluate the impact of alpha band activity on attentional capture, correlation coefficients between behavioral performance and alpha power were calculated. Specifically, the correlation between the alpha power contralateral to the items 200 ms before the onset of the search task and the capture effect of the item was analyzed. A full-sample Pearson’s correlation revealed a significant positive relationship for the cued item (r=0.43, p=0.03). To assess robustness, we conducted leave-one-out cross-validation, which yielded positive correlation coefficients across all 24 iterations (range: 0.183–0.497, mean r=0.430 ± 0.055), indicating that no single participant drove the effect. However, the Spearman rank correlation, which is more robust to univariate outliers, was not significant (r=0.13, p=0.57), and the p-values from the leave-one-out analysis varied considerably (range: 0.0158–0.4025). Taken together, these results suggest a positive trend that is directionally robust but whose statistical significance is sensitive to sample composition. Thus, we interpret this as preliminary evidence that occipital alpha activity may be associated with the priority state within VWM, warranting replication in larger samples. With this caveat, our findings broadly align with prior work linking alpha oscillations to working memory guidance (de Vries et al., 2017; de Vries et al., 2019).

**Figure 7.**
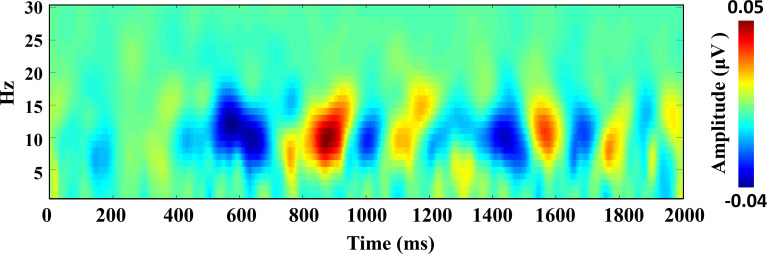
Time-frequency results, showing the difference in contralateral electrode activity (PO7/PO8) for same-color and same-location items under cued and uncued conditions.

Analysis of power differences between cued and uncued items unveiled a distinct pattern of alpha inhibition followed by rebound activation within the first 200 ms, as illustrated in Figure 7. Remarkably, this alternation in alpha band power persisted throughout the memory retention phase and showed a significant rhythm of approximately 4 Hz (Figure 9A, p<0.05).

**Figure 8.**
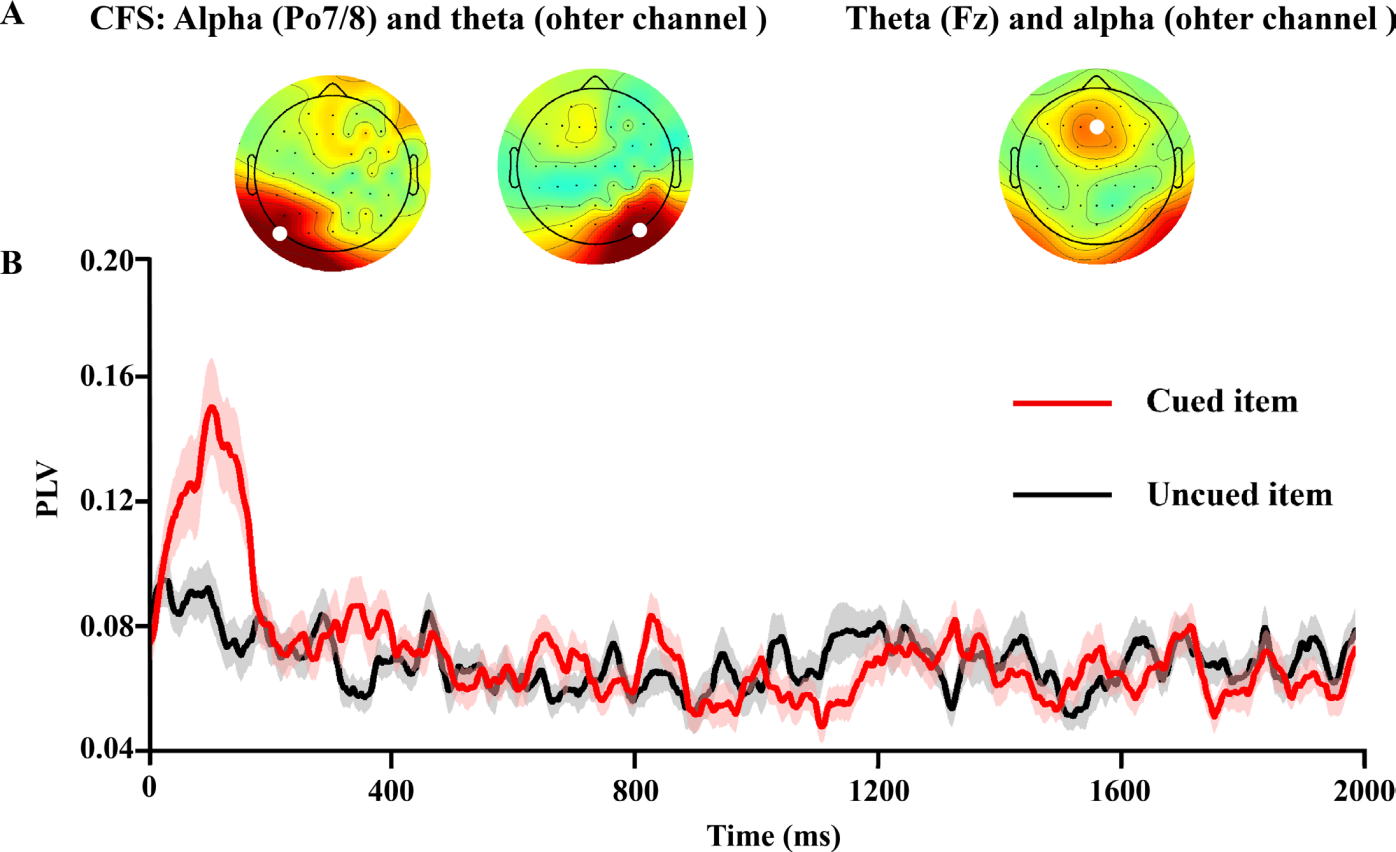
Fronto-occipital alpha-theta cross-frequency coupling and its alternating pattern during memory retention. (**A**) Topographical maps. 1:2 cross-frequency phase synchrony (CFS) between the alpha phase at the white electrode points and the theta phase at other electrode points (the left two maps), and the theta phase at the white electrode points and the alpha phase at other electrode points (the right map) during the memory retention phase. (**B**) Functional connectivity map. 1:2 CFS phase coupling between the alpha phase at the contralateral electrodes (PO7/PO8) of the two memory items (red lines represent the cued item; black lines represent the uncued item) and the theta phase at the frontal electrode (Pz) during the memory retention phase.

**Figure 9.**
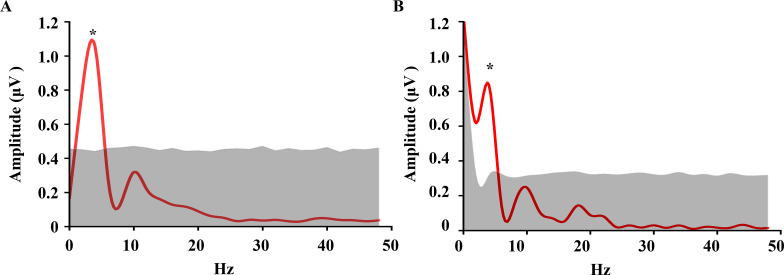
Spectrum analysis of alpha power and cross‑frequency coupling during memory retention. (**A**) Spectrum results. Power spectrum of the difference in contralateral alpha power between the two memory items during the retention phase across 0–50 Hz. (**B**) Spectrum results. Power spectrum of the difference in cross-frequency coupling (phase-locking value [PLV]-based 1:2 CFS) between the alpha phase at contralateral electrodes (PO7/PO8) and the theta phase at the frontal electrode (Fz) across 0–50 Hz during the retention phase. The gray shaded area represents the 0–95th percentile range from the permutation test; *: p<0.05.

Notably, both this study and prior research have identified that the alpha band decodes the preferred memory template. Intriguingly, an observation common to our study and earlier work is that the alternating frequency of this preferred template aligns with the theta rhythm range (4–7 Hz). To explore the potential impact of theta oscillations in different brain regions on alpha oscillations in regions contralateral to the memorized items, we quantified 1:2 cross-frequency phase synchrony (CFS) using the PLV between theta (4–7 Hz) and alpha (8–14 Hz) oscillations. Specifically, for each electrode pair, we computed PLV_1:2_ = $\left|\frac{\mathrm{1}}{\mathrm{N}}\sum \exp (\mathrm{i}\mathrm{(2}{\theta }_{\mathrm{theta}}-{\theta }_{\mathrm{alpha}}))\right| $, following established methodologies (Siebenhühner et al., 2020; Palva et al., 2005). The findings, illustrated in Figure 8A, indicated significant coupling between PO7/8 and the prefrontal regions. Additionally, CFS analysis revealed significant coupling for theta oscillations in the prefrontal cortex (Fz) and alpha oscillations in the visual regions. Fascinatingly, as illustrated in Figure 8B, by assessing the alpha-theta coupling strength between the posterior regions contralateral to the two items (PO7/8) and the prefrontal regions (Fz) on a moment-to-moment basis during the memory retention period, we found that the coupling strength of the two items alternated in lead. To further define the temporal frequency characteristics of these alternations, Fourier transformation was applied to the differences in PLV associated with the two items. An amplitude spectrum analysis across frequencies from 1 Hz to 50 Hz (Figure 9B) identified a significant peak at 4 Hz (p<0.05, FDR corrected), underscoring the rhythmic interconnection between these neural activities (see Figure 9B).

## Discussion

This study employed the function of VWM as a preferred template to automatically capture attention and integrated EEG techniques to systematically investigate the mechanisms underlying the representation of two memory items that occurred simultaneously or in close temporal proximity during the memory retention phase. Our findings revealed several key insights. First, we observed that the ability of the memory items to capture attention alternated over time, displaying opposite trends (Experiment 1). This indicates that the preference for the memory items alternated rather than occurring simultaneously. Power analysis showed alternating rhythms within the 4–7 Hz range (Experiments 1–3), corresponding to theta oscillations. Second, during the memory retention phase, the strength of alpha band oscillations, induced by cued and uncued items, alternated in dominance throughout the retention period. Third, we observed strong rhythmic alternations in the coupling strength between the alpha band of both posterior regions and the theta band of the prefrontal region. The coupling exhibited a leading frequency of approximately 4 Hz. In conclusion, our findings suggest that memory is maintained by alternately processing multiple items within the theta rhythm, rather than continuously entangling a single object. Furthermore, the posterior alpha oscillations reflect the prioritization of memory items and exhibit a close association with theta oscillations in the prefrontal cortex.

Traditional research on working memory has often adopted a static viewpoint, positing that its performance remains constant throughout the retention phase. This perspective, however, is challenged by the findings of the present study, which investigates the ability of two memory items to capture attention under various SOA conditions. Interestingly, the study reveals that the attentional capture abilities of these memory items alternate in dominance. Spectral analysis of the SOA function identified significant rhythmic fluctuations at 7 Hz, with opposing trends. This pattern suggests that, in scenarios requiring the maintenance of two working memory items for a task, these items alternately serve as the dominant template during the retention phase.

This discovery aligns with the existing literature on the rhythmic processing of external stimuli. Specifically, it has been well documented that attention oscillates within the theta rhythm (4–8 Hz) across various task types, including those based on spatial attention (Jensen and Mazaheri, 2010; Jiang et al., 2024), feature attention (Re et al., 2019), and object attention (Fiebelkorn et al., 2018; Fiebelkorn et al., 2013; Helfrich et al., 2018). Moreover, rhythmic attentional modulation has been observed in auditory attention (Ho et al., 2019; Ho et al., 2017), in the effects of top-down predictions on brightness perception (Han and VanRullen, 2017), and in the process of visual feature binding (Nakayama and Motoyoshi, 2019). These findings collectively highlight that attention operates within a theta rhythm, extending beyond external stimuli to include the internal processing of memory information (Peters et al., 2021; Pomper and Ansorge, 2021).

Similar to the present study, Peters et al., 2021, had participants memorize four spatial positions forming the endpoints of two objects (one cued), and their results showed that after the two objects disappeared, attention fluctuated at the theta rhythm between their original positions with an inverse correlation; in contrast, the present study explores the manner of memory maintenance indirectly by leveraging the guiding effect of working memory on attention, effectively avoiding the influence of spatial positions—while Peters et al.’s study, which directly examined differences in probe positions, clearly demonstrates that attention undergoes rhythmic changes at the two spatial locations and persists after the objects vanish, it hardly clarifies the rhythmicity of working memory performance, whereas the present study directly investigates such performance using the attention-capture effect of working memory, revealing that when maintaining multiple memory items, their attention-capturing capabilities alternate in dominance, i.e., multiple working memory items alternately become priority templates in a rhythmic manner.

The concept of a dynamic template mechanism emerges as a novel resolution to the ongoing debate between the single-template and multi-template hypotheses in working memory research (Frătescu et al., 2019; Olivers et al., 2011; van Moorselaar et al., 2014). This mechanism proposes that when maintaining multiple memory items, an individual represents these items through a single, dynamically shifting template, with each moment guided by a single item—a process that is spontaneous and not triggered by a retro-cue prompting a specific memory item. As observed in my Study 2, even in the presence of retro-cues, multiple memory items still begin with a particular item (consistent with individual habits) and sequentially become priority templates at the theta rhythm. This flexible approach not only aligns with the observed phenomenon of multiple memory items capturing attention, but also provides a new perspective on the discourse on how working memory operates, bridging a critical gap in the existing literature.

The current study reveals that during the memory retention phase, the dominance of posterior lateralized alpha oscillation alternates between two memory items and significantly correlates with behavioral outcomes. This finding aligns with extensive evidence, suggesting that alpha oscillations are crucial in modulating visual processing, going beyond mere sensory involvement to encompass functional roles in sensory gating and top-down control of preparatory activity (Klimesch, 2012; Jensen and Mazaheri, 2010; Chen et al., 2023; Gazzaley and Nobre, 2012). Notably, alpha band activity modulation is essential for prioritizing both incoming sensory data and stored memory content (Gresch et al., 2025; Van Diepen et al., 2019). A seminal example is a study in which subjects memorized two sequentially presented items, demonstrating the capability of posterior lateralized alpha oscillations to differentiate the memory item relevant to the current task, thereby elucidating the role of oscillations in managing the priority of concurrent task-relevant items (de Vries et al., 2019).

It’s critical to acknowledge that the investigation of posterior alpha oscillations in working memory, including the design of Experiment 3 in this study, often involves spatial differentiation of items (de Vries et al., 2019). Such spatial segregation allows alpha power modulations to be attributed to specific items through lateralization, achieved by presenting items in distinct hemifields. This methodological choice, driven by the spatial specificity of neurons encoding working memory items, serves as a pragmatic experimental strategy rather than implying that memory item prioritization solely depends on alpha oscillation lateralization (Foster et al., 2017; Gresch et al., 2024). For example, de Vries et al., 2017, discovered that posterior, rather than lateralized, alpha oscillations could delineate memory items related to forthcoming tasks.

The findings of this study, revealing strong phase coupling between posterior alpha oscillations and theta oscillations in the prefrontal lobe, contribute to the expanding literature on the intricate interplay between different brain oscillation frequencies and their roles in cognitive functions. The association of prioritization processes with theta oscillations in the frontal cortex is well documented (de Vries et al., 2020; de Vries et al., 2019; Quentin et al., 2019; Wallis et al., 2015), underscoring the critical role of the frontal cortex in orchestrating goal-directed behavior (Miller and Cohen, 2001), managing multiple objectives (Mansouri et al., 2017), and facilitating task switching (Monsell, 2003).

Particularly during working memory tasks, the frontal cortices play a critical role in processing and maintaining abstract goal-related representations and task-specific information. This is supported by the evidence of mixed selectivity in frontal neurons (Fusi et al., 2016) and their coordination of sensory areas based on this information (Christophel et al., 2017; Sreenivasan et al., 2014). For instance, fMRI studies have highlighted enhanced connectivity between frontal regions and posterior task-related sensory areas specific to working memory representations and those prioritized by maintenance cues (retrospective cues; Nelissen et al., 2013; van Ede and Nobre, 2023), with this increased connectivity correlating with improved performance of cued memory representations (Gazzaley and Nobre, 2012; Kuo et al., 2011).

Such evidence underscores the ability of the frontal cortex to selectively coordinate the activation of visual cortical working memory representations relevant to the current task. As outlined in a review by de Vries et al., 2020, alpha modulation facilitates the flexible tracking of changes in the state of VWM. This top-down control over the current state of VWM is accomplished through prefrontal theta oscillations, which in turn orchestrate alpha oscillations via a broad spectrum of cross-frequency interactions, highlighting a sophisticated mechanism of cognitive control and memory prioritization.

Although the present study successfully revealed the phenomenon of dynamic attentional templates through high-density behavioral sampling and preliminarily explored the underlying mechanisms using high-temporal-precision EEG technology—specifically, that prefrontal theta oscillations and visual cortical alpha oscillations play indispensable roles in this process—there are still some limitations. First, despite its high temporal resolution, the EEG technique used in this study makes it difficult to precisely localize the brain regions influencing dynamic templates due to the brain’s volume conduction effect (van den Broek et al., 1998). Second, the sample size of the current study is small, which may render the results vulnerable to interference from extreme cases (Button et al., 2013). Additionally, to assess cross-frequency interactions, we used 1:2 CFS quantified by the PLV (Siebenhühner et al., 2020; Palva et al., 2005; Siebenhühner et al., 2016). This measure directly captures the stability of the harmonic phase relationship (2θ_theta_ – θ_alpha_) and is robust against volume conduction because the two frequencies are distinct. The PLV of the two items alternately dominated over time, consistent with the pattern of behavioral data, which is hardly explicable by coincidence. Future research could employ more precise techniques (e.g. MEG devices) to accurately capture activities in different brain regions while maintaining high temporal precision. Furthermore, rhythmic transcranial magnetic stimulation could be used to modulate rhythms in various brain regions, altering the oscillation frequencies of working memory to provide causal evidence for this phenomenon.

Another limitation concerns the brain-behavior correlation between alpha power and attentional capture. Although the full-sample Pearson’s correlation was significant and leave-one-out analyses confirmed a consistently positive direction, the Spearman correlation was not significant, and the p-values of leave-one-out iterations varied considerably. This pattern likely reflects the modest sample size (N=24) and a moderate true effect size. Accordingly, the observed correlation should be considered preliminary, and future studies with larger samples are required to confirm whether alpha power reliably predicts the magnitude of working-memory-guided attentional capture.

In conclusion, our study aligns with the evolving understanding that oscillatory neural mechanisms underpin the complex interplay between attention and working memory. It builds on the premise that external attentional processes are intricately linked with neural oscillations, while also highlighting the crucial role of internal attention in modulating working memory representations through rhythmic activity. Our findings support a framework in which internal attention is mediated by a dynamic interaction between prefrontal theta oscillations, serving as a top-down control mechanism, and posterior alpha oscillations, which are instrumental in the selective inhibition or facilitation of memory items. This synthesis not only challenges conventional static views of working memory but also proposes a refined model of cognitive processing, where memory maintenance and attentional prioritization are orchestrated within a rhythmic neural symphony.

## Materials and methods

### Participants

A total of 25 participants (9 males, aged 22±1.9 years) took part in Experiment 1, 17 subjects (6 males, aged 19±1.5 years) participated in Experiment 2, and 27 subjects (12 males, aged 21±2.0 years) were involved in Experiment 3. All participants had normal or corrected-to-normal vision and no history of psychiatric or neurological disorders. The experiments were conducted in accordance with the Declaration of Helsinki and received ethical approval from the Research Ethics Committee at South China Normal University (approval date: 2021-04-01). Before the start of the experiment, all participants provided written informed consent. Participants completed the experiment in a dark, quiet, and isolated room, with their heads fixed on a headrest and their eyes looking directly at the center of the screen at a distance of 57 cm from the display.

### Stimuli

The same stimuli were used for all three experiments. The memory stimuli were a square in 6 colors: cyan (RGB: 5, 200, 200), red (RGB: 200, 80, 40), yellow (RGB: 200, 200, 5), blue (RGB: 5, 200, 200), purple (RGB: 112, 48, 160), and green (RGB: 50, 200, 50), each with a visual angle of 1.2°×1.2°. The search stimuli were colored outlined squares (1.2°×1.2°, 0.2° line thickness), with a 1.2° gap on the top, bottom, left side, or right side. The color of the search stimulus was consistent with that of the memory stimulus. The cue stimulus is a black square with side length 1.2°. The shape and color of the detection stimulus are consistent with those of the memory stimuli.

The experiments were programmed using the Psychtoolbox in MATLAB 2019b. All stimuli were presented on a 17-inch CRT monitor with a resolution of 1024×768, a refresh rate of 60 Hz, and a gray background (RGB: 128, 128, 128).

### Tasks

#### Experiment 1

Each trial began with a 500 ms fixation point, followed by a 1000 ms memory array. In the memory array, two differently colored memory stimuli were presented to the left and right at a distance of 3° from the center of the screen. After a 250 ms fixation point, the cue array was presented for 50 ms, with the cue appearing randomly on the left or right at a distance of 3° from the center of the screen. After a pseudo-random SOA (233 ms:33 ms:867 ms), either the search array (80% of trials) or the memory detection array (20% of trials) was presented for 2500 ms. In the search array, two search stimuli were presented directly above or below the screen center, 6° away from it; one had a gap facing up or down (the target), and the other had a gap facing left or right (the distractor). Participants were instructed to respond by pressing the ‘A’ key (for an upward-facing gap) or the ‘Z’ key (for a downward-facing gap). In the memory detection array, a detection stimulus was presented at the screen center for 2500 ms. Participants were asked to press the ‘N’ key (if it belonged) or the ‘M’ key (if it did not belong) to determine whether the detection stimulus belonged to either of the memory stimuli in the memory array, regardless of the cue. After responding, participants moved on to the next trial, with a 1000 ms interval between trials. To familiarize participants with the task, 15 practice trials were conducted before the formal experiment. Participants completed 30 blocks over 4 days within 1 week, with each block containing 150 trials (120 trials for search tasks and 30 trials for memory detection tasks).

#### Experiment 2

Each trial began with a 500 ms fixation point, followed by a 1000 ms memory array. In the memory array, two memory stimuli of different colors were presented on the left and right sides, 3° away from the center of the screen. After a pseudo-random SOA (233 ms:33 ms:867 ms), either the search array (80% of trials) or the memory detection array (20% of trials) was presented for 2500 ms. Participants’ tasks in the search array and memory detection array were consistent with those in Experiment 1. Over the course of 1 week (completed across 4 days), participants finished 30 blocks, with each block consisting of 150 trials (120 for search tasks and 30 for memory detection tasks).

#### Experiment 3

In Experiment 3, high-temporal-precision EEG technology was employed, which eliminated the need for dense SOA manipulation when investigating dynamic features. Additionally, to enable a longer memory retention time window during analysis, the interval between the cue array and the search array was fixed at 2000 ms. Furthermore, to encourage active maintenance of memory content during the retention phase, the interval between the cue array and the memory detection array was randomly varied between 200 ms and 2000 ms. Consistent with Experiment 1, 20% of the trials involved the memory detection array, and 80% involved the search array. The tasks for both the memory detection array and the search array were the same as in Experiment 1, and both were presented for 2500 ms. Participants were required to complete 4 blocks, with each block consisting of 150 trials.

### EEG recordings and preprocessing

EEG data was recorded using a cap with 64 electrodes arranged according to the international 10–20 system (Brain Products, Munich, Germany). The frontal electrode FCz was utilized as the online reference point, and the AFz electrode was employed as the ground. All electrodes were amplified using a 0.01–70 Hz online band-pass filter and continuously sampled at a rate of 1000 Hz per channel.

The offline continuous EEG data was preprocessed using EEGLAB, an open-source toolbox within the MATLAB environment. Initially, re-reference was conducted by using the bilateral mastoids TP9 and TP10. Next, all EEG signals underwent a 0.1 Hz high-pass filter, a 30 Hz low-pass filter, and a 50 Hz notch filter. Subsequently, independent component analysis was applied to each participant’s data to eliminate components related to eye movements and artifacts. The remaining components after this process were then projected back into the channel space. We extracted data from –500 ms to 2000 ms relative to cue stimulus presentation in Experiment 3.

### Data analysis

#### Behavioral performance analysis

We utilized the difference in reaction times (ΔRT) between the invalid trials (where the color of the memory item matched that of the interfering item) and the valid trials (where the color of the memory item matched that of the target item) extracted from the search array as the capture effects.

Subsequently, we employed a one-sample t-test to separately examine whether the memory accuracy rates of the two memory items in the three experiments were significantly higher than the guessing level (50%), and whether the capture effects of the two memory items were significantly greater than 0. In this process, the memory accuracy rates and capture effects in Experiment 1 and Experiment 2 were the results of averaging all the SOA conditions.

For Experiment 1, we concentrated on exploring how the capture effect of the two memory items on attention evolved over time. To accomplish this, we carried out a repeated-measures analysis of variance (ANOVA) with a 2 (cued item vs. uncued item)×20 (all SOAs) design. Post hoc comparisons were conducted using paired-sample t-tests to thoroughly investigate the potential changes in the capture effect of the memory items on attention.

In order to obtain the frequency spectrum of memory-based attention capture over time, we analyzed the difference in capture effects between the cued and uncued items across all SOAs at evenly spaced temporal intervals. Subsequently, we performed a fast Fourier transform (FFT) to estimate the spectral composition, which yielded power values across 14 frequency bins ranging from 1 Hz to 14 Hz. Regarding the phase relationship of 7 Hz oscillations between the cued and uncued items, we conducted separate Fourier transforms for these conditions as previously described. Then, we calculated the angular difference between the phase angles of the 7 Hz oscillations for each condition. This angular difference was projected onto the unit circle in the complex plane and averaged across all participants. The length and angle of the resulting vector represented the PLV (Lachaux et al., 1999) and the mean phase difference. To evaluate the statistical significance, a non-parametric approach was adopted to estimate the probability of the observed data under the null hypothesis. In 1000 permutation samples, each time course was shuffled before the analysis, generating one mean amplitude spectrum for the memory-based capture effect, and one mean phase difference between the cued and uncued conditions for each permutation sample. To control the false discovery rate at 5%, the individual frequency p-values in the amplitude spectrum were adjusted according to the number of frequency bins, following the method of Benjamini and Hochberg, 1995.

For Experiment 2, since Experiment 2 adopted a modified approach due to the absence of retro-cues. In this experiment, we compared the left and right memory items instead of the cued/uncued items. The item-based attentional benefit was calculated for each participant by subtracting the right-item capture effect from the left-item effect at equidistant intervals (233–867 ms). Individual FFTs (1–15 Hz) were performed on these difference scores before conducting a grand average across all participants. To determine the significance thresholds, we generated null distributions by randomly shuffling each participant’s time series 1000 times, recomputing the FFTs for each permutation, and using the median amplitude as the chance baseline. A paired-samples t-test comparing the original amplitudes against this chance baseline revealed significant 7 Hz oscillatory power (p<0.05, FDR corrected), as depicted in Figure 4.

#### Time-frequency decomposition

We used the short-time Fourier transform function in MATLAB 2019b to Fourier transform the baseline-corrected segments to obtain the power information, where the frequency was set to 30 frequency points of equal length from 1 Hz to 30 Hz, and the sliding window was a 200 ms Hanning window. The frequency-specific power at each time point was calculated as the square of the amplitude of the complex signal resulting from the convolution, determined by the sum of the squares of the real and imaginary parts (Cohen, 2014). Based on findings from previous studies indicating that memory stimuli can be characterized by alpha oscillations in the contralateral occipital lobes (de Vries et al., 2017), we therefore extracted alpha band data (8–14 Hz) from PO7 and PO8. We separately averaged the power of the contralateral side for the cued and uncued items. As illustrated in Figure 8, to compare the power induced by cued items with that induced by uncued items during the memory retention phase, we extracted the contralateral power for each color during memory retention. The power when cued was subtracted from the power when uncued. Finally, we averaged the results across all memory colors and participants.

#### Interregional connectivity

We quantified 1:2 CFS using the PLV between theta (4–7 Hz) and alpha (8–14 Hz) oscillations, following established procedures (Siebenhühner et al., 2020; Palva et al., 2005). Specifically, for each pair of electrodes, we computed:

$$\displaystyle  \mathrm{C}\mathrm{F}\mathrm{S}_{1\colon 2}=\left|\frac{1}{\rm N}\sum {\rm exp}\left ({\rm i}\left (2\cdot \theta _{\mathrm{t}\mathrm{h}\mathrm{e}\mathrm{t}\mathrm{a}}\left (t\right)-\theta _{\mathrm{a}\mathrm{l}\mathrm{p}\mathrm{h}\mathrm{a}}\left (t\right)\right)\right)\right|,$$

where theta θ_theta_ and θ_alpha_ alpha are the instantaneous phases of theta and alpha oscillations, respectively. The factor 2 accounts for the 1:2 frequency ratio (theta:alpha = 1:2), effectively aligning the faster alpha oscillation to the slower theta rhythm (phase acceleration). The analysis first revealed that both electrodes PO7 and PO8 exhibited the strongest 1:2 CFS with electrodes in the prefrontal region. Next, we calculated the 1:2 CFS between theta oscillations at the frontal electrode Fz and alpha oscillations at all other electrodes to confirm the specificity of cross-frequency coupling between the prefrontal cortex and visual cortex. Finally, we extracted the 1:2 CFS values between the contralateral visual electrodes PO7/8 (relative to the cued and uncued items, respectively) and the frontal electrode Fz.

#### The correlation between neural activation and behavior

As shown in Figure 6, a clear activation of the alpha band was observed during the memory retention phase. To investigate whether this activation was related to behavioral performance, we calculated the alpha activation strength in the 200 ms preceding the search task. Specifically, we computed the average amplitude within the 8–14 Hz frequency range during the 1800–2000 ms time window of the retention phase, contralateral to the cue. This was then correlated with the capture effect size of the cued items using Pearson’s correlation. To assess the robustness of this relationship against potential outliers and sample composition, we additionally performed leave-one-out cross-validation and Spearman rank correlation. These complementary analyses are reported in the Results section.

#### Frequency spectrum analysis

As shown in Figure 8, the alpha power (8–14 Hz) induced by cued and uncued items alternated in dominance during the memory retention phase. To quantify this rhythmic alternation, we conducted a spectral analysis following these steps: First, we computed the power difference between cued and uncued items within the 8–14 Hz range during the retention phase. These differences were then downsampled to 100 Hz using a 10 ms window for averaging, generating a one-dimensional time series spanning the 0–2000 ms retention period. This time series was subsequently subjected to amplitude spectrum analysis across frequencies from 1 Hz to 50 Hz using Fourier transformation.

To assess the statistical significance of the observed spectral features, we employed a permutation test. Specifically, we randomly shuffled the temporal order of the time series of power differences between cued and uncued items—thereby preserving the amplitude distribution of the data while eliminating temporal correlations in the original sequence—and repeated the Fourier transform and spectral analysis for each shuffled time series. This permutation process was replicated 1000 times to generate a null distribution of spectral power values (Maris and Oostenveld, 2007; Delorme and Makeig, 2004). A frequency component in the original data was considered statistically significant if its power ranked within the top 5% of the corresponding null distribution (p<0.05).

We applied the same analytical pipeline to investigate differences in the PLV between the contralateral regions of the two items and the prefrontal cortex during the retention phase. Specifically, PLV differences (i.e. the difference between the two conditions) were computed, downsampled to 100 Hz using a 10 ms window for averaging to generate a time series spanning 0–2000 ms, and then subjected to amplitude spectrum analysis (1–50 Hz) using Fourier transformation. Significance was assessed via the identical permutation test procedure described above (randomly shuffling the temporal order of the difference time series).

## Data Availability

The raw and processed behavioral and EEG data, along with all analysis scripts necessary to reproduce the main findings, are publicly available at https://osf.io/34cex/. All analyses were conducted using custom code in MATLAB and the EEGlab toolbox. The following dataset was generated: LuJ 2025Visual working memory guides attention rhythmicallyOpen Science Framework34cex10.7554/eLife.108017PMC1334563242418336

## Additional files

MDAR checklist
