# Supplementary material for: Visual working memory guides attention rhythmically in humans
Source: eLife. 2026 Jul 8;14:RP108017. doi: 10.7554/eLife.108017 (PMC13345632; doi:10.7554/eLife.108017)
Supplement: Peer review history

# Editor report: eLife Assessment

**DOI:** 10.7554/eLife.108017.4.sa0

**Authors:** Shuo Wang

**Affiliations:** Washington University in St. Louis United States

**Keywords:** Solid, Valuable

This **valuable** study reports evidence that items maintained in working memory can bias attention in an oscillatory manner, with the attentional capture effect fluctuating at theta frequency. The study provides **solid** evidence that this dynamic attentional bias is associated with oscillatory neural mechanisms, particularly in the alpha and theta bands, as measured by EEG. The study will be relevant for researchers studying attention, working memory, and neural oscillations, particularly those interested in how memory and perception interact over time.

# Referee report: Reviewer #1 (Public review)

**DOI:** 10.7554/eLife.108017.4.sa1

Summary

In the presented paper, Lu and colleagues focus on how items held in working memory bias someone's attention. In a series of three experiments, they utilized a similar paradigm in which subjects were asked to maintain two colored squares in memory for a short and variable time. After this delay, they either tested one of the memory items or asked subjects to perform a search task.

In the search task, items could share colors with the memory items, and the authors were interested in how these would capture attention, using reaction time as a proxy. The behavioral data suggest that attention oscillates between the two items. At different maintenance intervals, the authors observed that items in memory captured different amounts of attention (attentional capture effect).

This attentional bias fluctuates over time at approximately the theta frequency range of the EEG spectrum. This part of the study is a replication of Peters and colleagues (2020).

Next, the authors used EEG recordings to better understand the neural mechanisms underlying this process. They present results suggesting that this attentional capture effect is positively correlated with the mean amplitude of alpha power. Furthermore, they show that the weighted phase lag index (wPLI) between the alpha and theta bands across different electrodes also fluctuates at the theta frequency.

Strengths

The authors focus on an interesting and timely topic: how items in working memory can bias our attention. This line of research could improve our understanding of the neural mechanisms underlying working memory, specifically how we maintain multiple items and how these interact with attentional processes. This approach is intriguing because it can shed light on neuronal mechanisms not only through behavioral measures but also by incorporating brain recordings, which is definitely a strength.

Subjects performed several blocks of experiments, ranging from 4 to 30, over a few days depending on the experiment. This makes the results - especially those from behavioral experiments 2 and 3, which included the most repetitions - particularly robust.

Comments on revision:

The authors have adequately addressed my concerns. No further comments.

# Author comment: Author response

**DOI:** 10.7554/eLife.108017.4.sa2

**Authors:** Jiachen Lu, Yaochun Cai, Xilin Zhang

**Affiliations:** Guangzhou University Guangzhou China; Key Laboratory of Brain, Cognition and Education Sciences, Ministry of Education, South China Normal University Guangzhou China; Key Laboratory of Brain, Cognition and Education Sciences, Ministry of Education, South China Normal University Guangzhou China

The following is the authors’ response to the previous reviews

**Public Reviews:**

**Reviewer #1 (Public review):**

Weaknesses

(1) One of the main EEG results is based on the weighted phase lag index (wPLI) between oscillations in the alpha and theta bands. In my opinion, this is problematic, as wPLI measures the locking of oscillations at the same frequency. It quantifies how reliably the phase difference stays the same over time. If these oscillations have different frequencies, the phase difference cannot remain consistent. Even worse, modeling data show that even very small fluctuations in frequency between signals make wPLI artificially small (Cohen, 2015).

In response authors stated : "Additionally, the present study referenced previous research by using the wPLI index as a measure of cross-frequency coupling strength31,64-66"

Unfortunately, after checking those publications, we can see that in paper 31 there is no mention of "wPLI" or "PLV." In 64 and 65, the authors use wPLI, but only to measure same-frequency coherence, whereas cross-frequency coupling is computed by phase-amplitude coupling or cross-frequency coupling also known as n:m-PS. In 66, I cannot find any cross-frequency results, only cross-species analysis. This is very problematic, as it indicates that the authors included references in their rebuttal without verifying their relevance.

31 de Vries, I. E. J., van Driel, J., Karacaoglu, M. & Olivers, C. N. L. Priority Switches in Visual Working Memory are Supported by Frontal Delta and Posterior Alpha Interactions. Cereb Cortex 28, 4090-4104, doi:10.1093/cercor/bhy223 (2018).

64 Delgado-Sallent, C. et al. Atypical, but not typical, antipsychotic drugs reduce hypersynchronized prefrontal-hippocampal circuits during psychosis-like states in mice: Contribution of 5-HT2A and 5-HT1A receptors. Cerebral Cortex 32, 870 3472-3487 (2022).

65 Siebenhühner, F. et al. Genuine cross-frequency coupling networks in human resting-state electrophysiological recordings. PLoS Biology 18, e3000685 (2020).

66 Zhang, F. et al. Cross-Species Investigation on Resting State Electroencephalogram. Brain Topogr 32, 808-824, doi:10.1007/s10548-019-00723-x (2019).

We thank the reviewer for this critical methodological correction. We fully agree that the weighted phase lag index (wPLI) is designed for same-frequency phase synchronization and is not appropriate for cross-frequency coupling (CFC). In our original rebuttal, we incorrectly cited references that did not support the use of wPLI for CFC. We apologize for this error and have thoroughly revised our analysis and manuscript.

What we have done:

(1) Replaced wPLI with proper 1:2 cross-frequency phase synchrony (CFS).

We now compute 1:2 CFS using the phase-locking value (PLV) between theta (4–7 Hz) and alpha (8–14 Hz) oscillations, following established methodologies (Siebenhühner et al., 2020, PLoS Biol; Palva et al., 2005, J Neurosci). Specifically, for each electrode pair we compute:

$\mathrm{CFS}_{1: 2}=\left\lvert\, \frac{1}{N} \sum \exp \left(i\left(2 \theta_{\text {theta}}-\theta_{\text {alpha}}\right)\right)\right.$.The factor 2 accounts for the 1:2 frequency ratio (theta:alpha = 1:2).

(2) Updated all relevant sections – Methods (“Interregional connectivity”), Results (Figure 8, Figure 9), Discussion, and Figure legends – replacing “wPLI” with “1:2 CFS (PLV)” and providing the correct formula and citations.

(3) Corrected the reference list to include the appropriate methodological papers (Siebenhühner et al., 2020; Palva et al., 2005) and removed irrelevant citations.

We believe this revision fully resolves the reviewer’s concern. Notably, the empirical results remained qualitatively unchanged (PLV and wPLI gave highly consistent values due to the absence of zero‑lag artifacts in cross‑frequency coupling), so the main conclusions of the paper are unaffected.

(2) Another result from the electrophysiology data shows that the attentional capture effect is positively correlated with the mean amplitude of alpha power. In the presented scatter plot, it seems that this result is driven by one outlier. Unfortunately, Pearson correlation is very sensitive to outliers, and the entire analysis can be driven by an extreme case. I extracted data from the plot and obtained a Pearson correlation of 0.4, similar to what the authors report. However, the Spearman correlation, which is robust against outliers, was only 0.13 (p = 0.57) indicating a non-significant relationship.

Cohen, M. X. (2015). Effects of time lag and frequency matching on phase based connectivity. Journal of Neuroscience Methods, 250, 137-146

We thank the reviewer for raising this important statistical issue. We have conducted a thorough robustness analysis and revised our interpretation accordingly.

What we have done:

(1) Removed the original scatter plot (Figure 7) to avoid overinterpretation. No replacement figure is provided; instead, all results are reported in text.

(2) Conducted leave‑one‑out cross‑validation.

The Pearson correlation remained positive across all 24 iterations (range: 0.183–0.497, mean r = 0.430 ± 0.055), confirming that no single participant solely drove the direction of the effect.

(3) Reported Spearman rank correlation (r = 0.13, p = 0.57), which is more robust to univariate outliers.

(4) Acknowledged the sensitivity – p‑values from leave‑one‑out iterations ranged from 0.0158 to 0.4025, indicating that statistical significance is not fully robust to sample composition.

(5) Revised the text to present this as preliminary evidence rather than a definitive conclusion. Specifically, we state: “Thus, we interpret this as preliminary evidence that occipital alpha activity may be associated with the priority state within VWM, warranting replication in larger samples.” The Discussion also includes a dedicated limitation paragraph.
